# Combining bioinformatics and machine learning to identify common mechanisms and biomarkers of chronic obstructive pulmonary disease and atrial fibrillation

**DOI:** 10.3389/fcvm.2023.1121102

**Published:** 2023-03-28

**Authors:** Ziyi Sun, Jianguo Lin, Tianya Zhang, Xiaoning Sun, Tianlin Wang, Jinlong Duan, Kuiwu Yao

**Affiliations:** ^1^ Guang’anmen Hospital, China Academy of Chinese Medical Sciences, Beijing, China; ^2^ Graduate School, Beijing University of Chinese Medicine, Beijing, China; ^3^ Graduate School, China Academy of Chinese Medical Sciences, Beijing, China; ^4^ Graduate School, Hebei University of Chinese Medicine, Shijiazhuang, China; ^5^ Eye Hospital China Academy of Chinese Medical Sciences, China Academy of Chinese Medical Sciences, Beijing, China

**Keywords:** chronic obstructive pulmonary disease, atrial fibrillation, microarray, machine learning, biomarker

## Abstract

**Background:**

Patients with chronic obstructive pulmonary disease (COPD) often present with atrial fibrillation (AF), but the common pathophysiological mechanisms between the two are unclear. This study aimed to investigate the common biological mechanisms of COPD and AF and to search for important biomarkers through bioinformatic analysis of public RNA sequencing databases.

**Methods:**

Four datasets of COPD and AF were downloaded from the Gene Expression Omnibus (GEO) database. The overlapping genes common to both diseases were screened by WGCNA analysis, followed by protein-protein interaction network construction and functional enrichment analysis to elucidate the common mechanisms of COPD and AF. Machine learning algorithms were also used to identify key biomarkers. Co-expression analysis, “transcription factor (TF)-mRNA-microRNA (miRNA)” regulatory networks and drug prediction were performed for key biomarkers. Finally, immune cell infiltration analysis was performed to evaluate further the immune cell changes in the COPD dataset and the correlation between key biomarkers and immune cells.

**Results:**

A total of 133 overlapping genes for COPD and AF were obtained, and the enrichment was mainly focused on pathways associated with the inflammatory immune response. A key biomarker, cyclin dependent kinase 8 (*CDK8*), was identified through screening by machine learning algorithms and validated in the validation dataset. Twenty potential drugs capable of targeting *CDK8* were obtained. Immune cell infiltration analysis revealed the presence of multiple immune cell dysregulation in COPD. Correlation analysis showed that *CDK8* expression was significantly associated with CD8+ T cells, resting dendritic cell, macrophage M2, and monocytes.

**Conclusions:**

This study highlights the role of the inflammatory immune response in COPD combined with AF. The prominent link between *CDK8* and the inflammatory immune response and its characteristic of not affecting the basal expression level of nuclear factor kappa B (*NF-kB)* make it a possible promising therapeutic target for COPD combined with AF.

## Introduction

Chronic obstructive pulmonary disease (COPD) is characterized by progressive airway obstruction. It is estimated that COPD will be the third most deadly disease in the world by 2030 (1). In clinical practice, COPD is often combined with multiple cardiovascular diseases, including heart failure, coronary atherosclerotic heart disease, and atrial fibrillation (AF) (2). AF, the most common type of arrhythmia, has been shown in epidemiological surveys to affect at least 33.5 million people worldwide (3). Heart failure and stroke are severe complications of AF, and the risk of heart failure in AF patients is about twice as high as normal, and the risk of stroke is 4–5 times higher (4, 5).

There is a strong association between COPD and AF, with studies showing a 2.23-fold increased risk of AF in COPD patients compared to non-COPD patients (6). Similarly, the prevalence of COPD in patients with AF reached 25% (7). In addition, combined COPD increases the recurrence rate and the incidence of adverse events after catheter ablation in patients with AF (8, 9). A meta-analysis that included 46 studies involving 4,232,784 AF patients showed that AF patients with comorbid COPD had a significantly increased risk of bleeding, cardiovascular event death, and all-cause mortality compared to AF patients without COPD (10). It is currently thought that enhanced sympathetic activity, altered cardiac structure, immune dysfunction, inflammation, and oxidative stress may be involved in the development of AF in patients with COPD. However, the exact mechanisms have not been fully elucidated (11). Meanwhile, drugs commonly used to treat COPD, such as beta-blockers, theophylline, and glucocorticoids, have been linked to an increased risk of AF development (12, 13). Therefore, it is of practical clinical significance to explore the potential mechanisms of COPD and AF co-morbidity at the genetic level and to find promising therapeutic targets for application.

**Figure 1:**
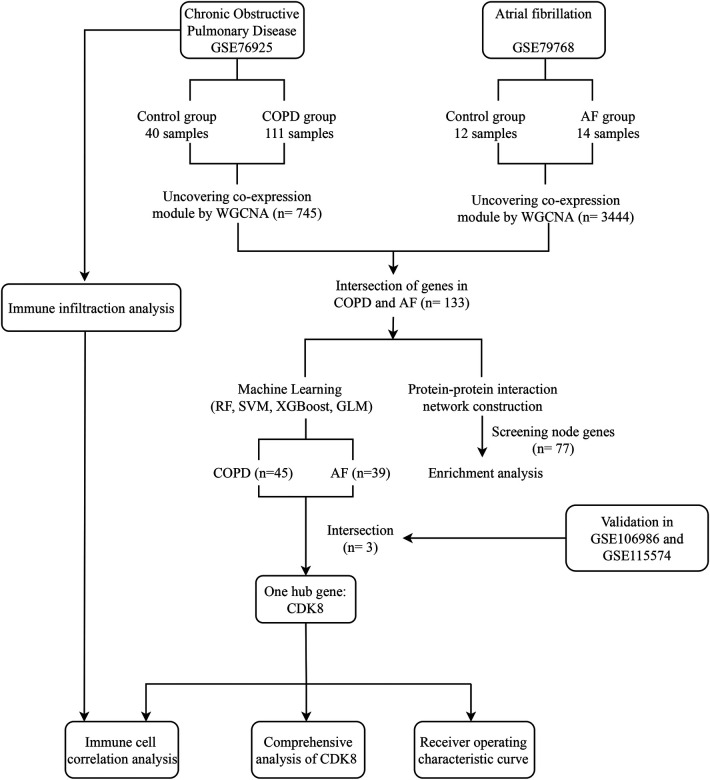
Study flowchart. GSE, gene expression omnibus series; WGCNA, weighted gene co-expression network analysis; RF, Random forest support vector machine; SVM, support vector machine; XGBoost, extreme gradient boosting; GLM, generalized linear model.

The field of bioinformatics is developing rapidly, and large amounts of genetic data are publicly available to uncover many unknown pathophysiological mechanisms in the development of diseases and potential connections between diseases. Machine learning, an essential artificial intelligence component, has also been widely applied to bioinformatics research and has become an important tool (14). Based on this, this study integrates COPD and AF mRNA datasets in public databases and attempts to reveal the common biological mechanisms of COPD and AF co-morbidity through weighted gene co-expression network analysis (WGCNA), protein-protein interaction (PPI) network construction, and enrichment analysis. Random forest (RF), support vector machine (SVM), extreme gradient boosting (XGBoost), and generalized linear model (GLM) were used to screen potential biomarkers. A comprehensive analysis of key biomarkers was performed, including co-expression analysis, construction of “transcription factor (TF)-mRNA-microRNA (miRNA)” regulatory networks, and drug prediction. An examination of immune cell infiltration on the COPD dataset was also carried out. Figure 1 depicts the study flowchart.

## Materials and methods

### Data source

We applied the Gene Expression Omnibus (GEO) database (15) (http://www.ncbi.nlm.nih.gov/geo/) to filter gene expression datasets for microarrays by qualifying the keywords “COPD” and “AF” with the filter criteria “Homo Sapiens” and “tissues.” Four datasets were finally obtained. In the COPD group, GSE76925 (lung tissue samples from 111 COPD patients and 40 control patients) (16) and GSE106986 (lung tissue samples from 14 COPD patients and 5 control patients) were selected. In the AF group, GSE79768 (left and right atrial tissue samples from 7 AF patients and 6 control patients) (17) and GSE115574 (left and right atrial tissue samples from 15 AF patients and 15 control patients) (18) were selected. GSE76925 and GSE79768 were used as training sets, GSE106986 and GSE115574 were used as the external validation set.

### Weighted gene co-expression network analysis

WGCNA analysis was performed on GSE76925 and GSE79768, respectively, to obtain modules closely associated with COPD and AF. WGCNA analysis was constructed using the “WGCNA” package in R (19). The genes were ranked based on the standard deviation of gene expression. The top 25% of genes with the largest fluctuations were selected for subsequent analysis, and outlier samples were excluded by hierarchical clustering. The R2 was set greater than 0.9, and a suitable soft threshold (β) was calculated to make the network conform to the scale-free distribution. The co-expression modules are identified by hierarchical clustering to obtain a hierarchical clustering tree. Finally, the module feature values and the correlation between module feature values and clinical features are calculated to obtain the expression spectrum of each module, which is expressed by the correlation coefficient as well as the *p*-value. Finally, we select the genes in the modules closely related to the disease for subsequent analysis.

### Identification of overlapping genes and PPI network analyses

The genes in the modules closely related to disease in GSE76925 and GSE79768 obtained by WGCNA analysis were taken to intersect. The Venn diagram was used to visualize the overlapping genes. After that, the overlapping genes were imported into the “Search Tool for Interacting Genes” (STRING) online platform (https://cn.string-db.org/) (20). The species was limited to “Homo sapiens,” with the conﬁdence score set to an intermediate value (conﬁdence score > 0.4) to construct the PPI network. The results were exported to Cytoscape 3.7.2 for visualization (21).

### Functional enrichment analysis

To further understand the common biological mechanisms between the two diseases, the protein information in the PPI network was enriched for Gene Ontology (GO) and Kyoto Encyclopedia of Genes and Genomes (KEGG) pathways using the “clusterProfiler” package in R (22). The GO includes biological process (BP), cellular component (CC), and molecular function (MF) (23). The screening condition was set at adjust *-P* *<* 0.05, and the visualization was presented using the Sangerbox platform (http://vip.sangerbox.com/). Meanwhile, Gene set enrichment analysis (GSEA) enrichment analysis was performed on the GSE76925 and GSE79768 datasets to comprehensively analyze the key pathways associated with COPD and AF pathogenesis. The reference dataset was “c5.kegg.v7.4.symbols.gmt” from the MSigDB database (24). The significantly enriched pathways were identified with the screening criteria of *P <* 0.05 and FDR < 0.25, and the “enrichplot” package was used for visualization.

### Identification of candidate genes based on machine learning algorithms

Machine learning is now widely used to identify characteristic genes. To identify key genes associated with COPD and AF, four machine learning algorithms, including RF, SVM, XGBoost, and GLM, were used to screen for key genes in COPD and AF, respectively. In both training datasets, the response variable was set to whether the diagnosis was COPD and AF, and the overlapping genes were set as explanatory variables. Use 70% of the data for model construction and 30% for model validation. Models for RF, SVM, XGBoost, and GLM were constructed separately using the “caret” R package (25). It is well known that while building a machine learning model with good results, it is equally important to evaluate the interpretation of the model, as only an interpretable machine learning model is likely to be more widely understood and adopted. The “DALEX” R package is a model interpretation package that has been developed to help understand the links between input variables and model outputs (26). It uses the size of the residuals to assess the quality of the model (smaller residuals mean better model quality) and the root mean square error (RMSE) to assess the importance of the variables (defined as how much the absence of a variable affects the predicted value of the response variable). Once modelling was completed, residual box plots were drawn for the four models using the “DALEX” R package and the RMSE was used to assess the importance of each gene in the model. Also, we use the “predict” function in R to verify the accuracy of the predictions of the model constructed by “caret”. Receiver operating characteristic (ROC) curves were then plotted using the “pROC” R package (27) and the area under the curve (AUC) was reported to assess the predictive effectiveness of models. Finally, we selected models with high predictive accuracy based on the quality of the model (assessed by the size of the residuals) and the area of the AUC. According to the gene importance score, select the top 20 genes from the constructed models for COPD and AF respectively, and then perform the intersection for these genes. Afterwards, we compared the differential expression of intersecting genes between the two datasets, with *P* < 0.05 considered to be significantly different, and used the “ggpubr” R package to plot boxplots. Finally, genes that were differentially expressed in the disease and control groups in both sets of data and that showed the same expression trend in both microarrays were identified as candidate hub genes.

### Validation of hub genes and evaluation of prediction accuracy

The identified candidate genes were validated in the validation sets for COPD and AF, respectively. The comparison of gene expression between disease group and control group with *P *< 0.05 was considered to be significantly different. The candidate genes with significant differences were finally considered to be hub genes and the boxplot was drawn for visualization. Afterwards, ROC curves of the diagnostic value of hub genes in the training set and validation set were plotted using “pROC” R package, and AUC was calculated to evaluate the accuracy of hub gene prediction.

### Comprehensive analysis of hub genes

Hub genes were entered into the GeneMANIA online website (28) (http://genemania.org) for co-expression and functional enrichment analyses. The JASPAR database (29) (http://jaspar.genereg.net/) were used to predict TFs regulating hub genes. Prediction of miRNAs regulated by hub genes using the miRTarBase database (30) (https://mirtarbase.cuhk.edu.cn/), and experimentally validated miRNAs were selected. The results were visualized using Cytoscape to demonstrate the “TF-mRNA-miRNA” regulatory network. In addition, we used the DGIDB 3.0 database (31) (http://www.dgidb.org/) to predict potential drugs that could target the hub gene.

### Immune cell inﬁltration analysis

CIBERSORT can calculate the proportion of different immune cells in the gene expression profile through a deconvolution algorithm (32). We performed an immune cell infiltration analysis of the COPD gene expression matrix (GSE76925) using the CIBERSORT algorithm. The “barplot” and “vioplot” packages were used to show the relative proportions and differences of immune cell types in the expression profile between the control and COPD groups. The “corrplot” package was used to show the correlation heat map of immune cells in the expression profile. The hub gene's expression was then taken from the expression profile. The correlation between the hub gene and immune cells was analyzed by Spearman correlation analysis, with *P* < 0.05 as the screening condition, and visualized by the “ggplot” package.

## Results

### Construction of co-expressed gene modules

**Figure 2:**
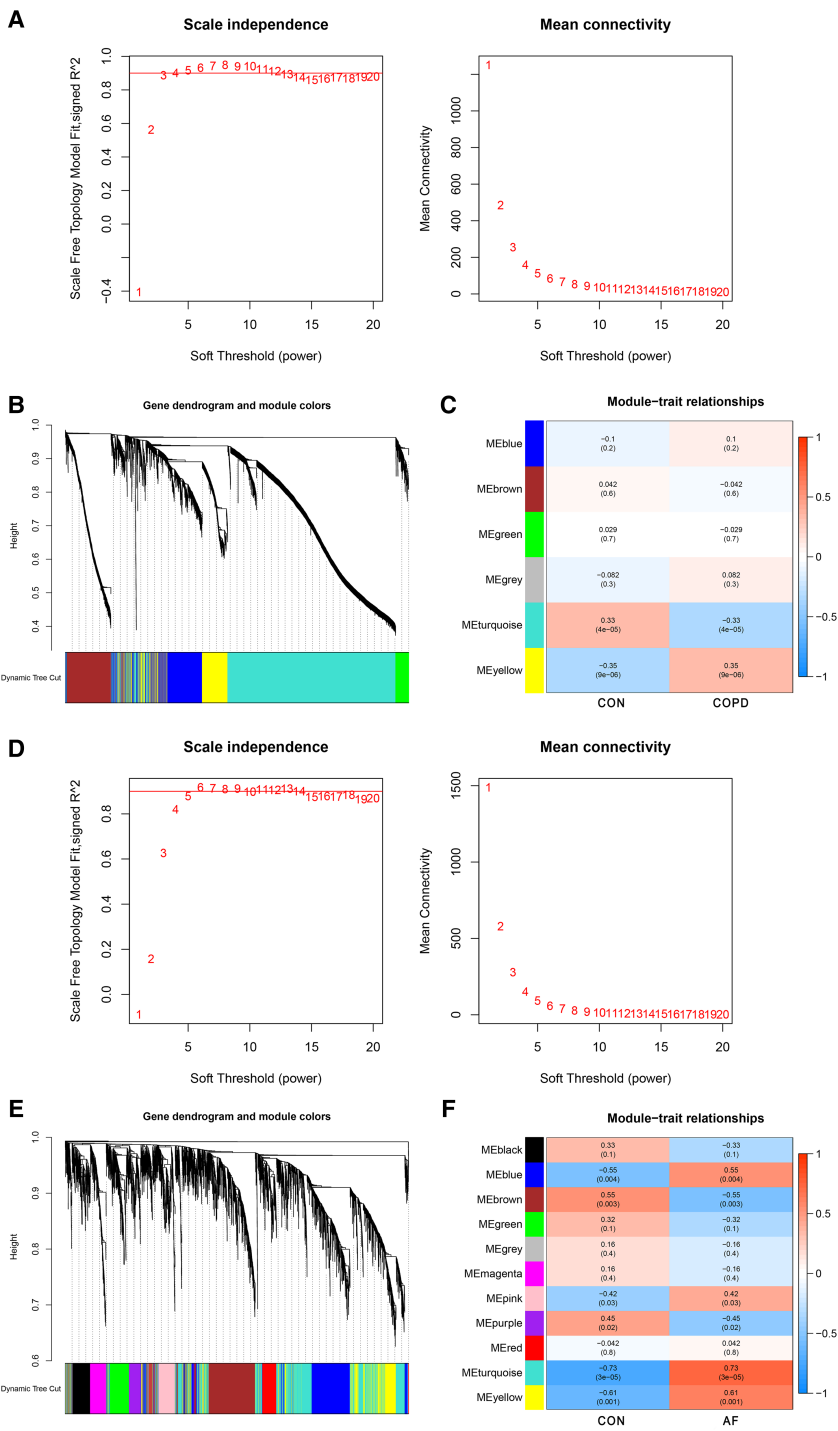
Identification of COPD and AF module genes *via* WGCNA. (**A**) The scale-free fit index for soft-thresholding powers and mean connectivity (GSE76925). (**B**) Dendrogram of the genes clustered (GSE76925). (**C**) Module-trait relationships heatmap (GSE76925). The numbers in each cell means the correlation coefficient and *p*-value. (**D**) The scale-free fit index for soft-thresholding powers and mean connectivity (GSE79768). (**E**) Dendrogram of the genes clustered (GSE79768). (**F**) Module-trait relationships heatmap (GSE79768).

WGCNA analysis was performed on the GSE76925 and GSE79768 datasets to identify co-expression modules associated with COPD and AF, respectively. The β selection analysis of GSE76925 showed that the network was closer to the scale-free network when the *β* = 4 (Figure 2A). Six modules were also identified, of which the yellow module was positively associated with COPD (correlation coefficient = 0.35, *P* = 9e-06) and contained 745 genes (Figures 2B,C). The β selection analysis of GSE79768 showed that the network was closer to the scale-free network when the *β* = 10 (Figure 2D). Eleven modules were identified, among which blue (correlation coefficient = 0.55, *P* = 0.004), pink (correlation coefficient = 0.42, *P* = 0.03), turquoise (correlation coefficient = 0.73, *P* = 3e-05), and yellow (correlation coefficient = 0.61, *P* = 0.001) were positively correlated with AF and contained 3,444 genes (Figures 2E,F). Among them, 952 were blue modules, 287 were pink modules, 1,526 were turquoise modules, and 705 were yellow modules. Detailed gene information is listed in Supplementary Table S1.

### Identification of overlapping genes and construction of PPI network

**Figure 3:**
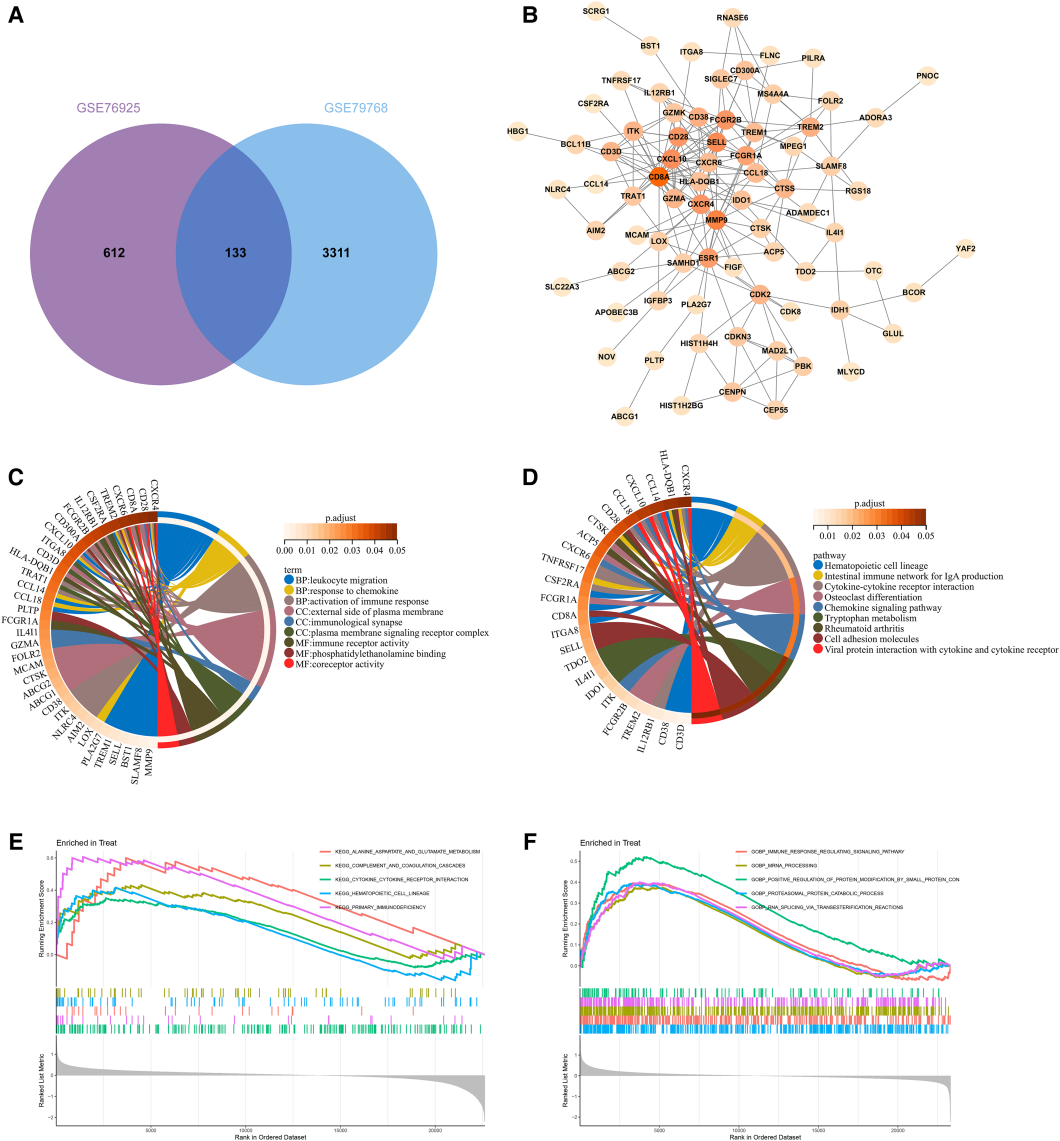
Construction of overlapping gene PPI networks and enrichment analysis. (**A**) Venn diagram of COPD and AF module genes. (**B**)PPI network. (**C**) KEGG pathway analysis of genes. Different colors represent various significant pathways and related enriched genes. (**D**) GO analysis of genes. Different colors represent various significant pathways and related enriched genes. (**E**) GSEA enrichment analysis of upregulated genes in the COPD dataset. (**F**) GSEA enrichment analysis of upregulated genes in the AF dataset.

By taking the intersection of the genes of COPD and AF-related modules obtained by WGCNA, 133 genes common to COPD and AF were obtained (Figure 3A). After that, we constructed a PPI network of overlapping genes and excluded genes that did not interact. Finally, we obtained 77 interacting genes in the network (Figure 3B).

### Functional enrichment analysis

77 genes in the PPI network were analyzed for GO and KEGG enrichment to reveal the underlying molecular biological processes shared between COPD and AF. The GO enrichment analysis revealed that 77 genes were primarily enriched in the “Leukocyte migration,” “Response to chemokine,” “Cellular response to chemokine,” “Myeloid leukocyte migration,” “Activation of immune response,” “Regulation of inflammatory response”(BP); “External side of plasma membrane,” “Immunological synapse,” “Plasma membrane signaling receptor complex,” (CC); “Immune receptor activity,” “Phosphatidylethanolamine binding,” “Coreceptor activity,” (MF) (Figure 3C, Supplementary Table S2). Also, KEGG analysis showed that genes were enriched in “Hematopoietic cell lineage,” “Intestinal immune network for IgA production,” “Cytokine-cytokine receptor interaction,” “Chemokine signaling pathway,” “Cell adhesion molecules,” “T cell receptor signaling pathway” (Figure 3D, Supplementary Table S3). GSEA enrichment analysis revealed that genes upregulated in the GSE76925 dataset were mainly enriched in signaling pathways such as “Cytokine-cytokine receptor interaction,” “Primary immunodeficiency,” “Alanine aspartate and glutamate metabolism,” “Hematopoietic cell lineage,” “Complement and coagulation cascade” (Figure 3E). The genes upregulated in the GSE79768 dataset were mainly enriched in “RNA degradation,” “Ubiquitin-mediated proteolysis,” “Spliceosome,” “Leukocyte transendothelial migration,” and “Natural killer cell-mediated cytotoxicity” (Figure 3F).

### Identification of hub genes based on machine learning algorithms

**Figure 4:**
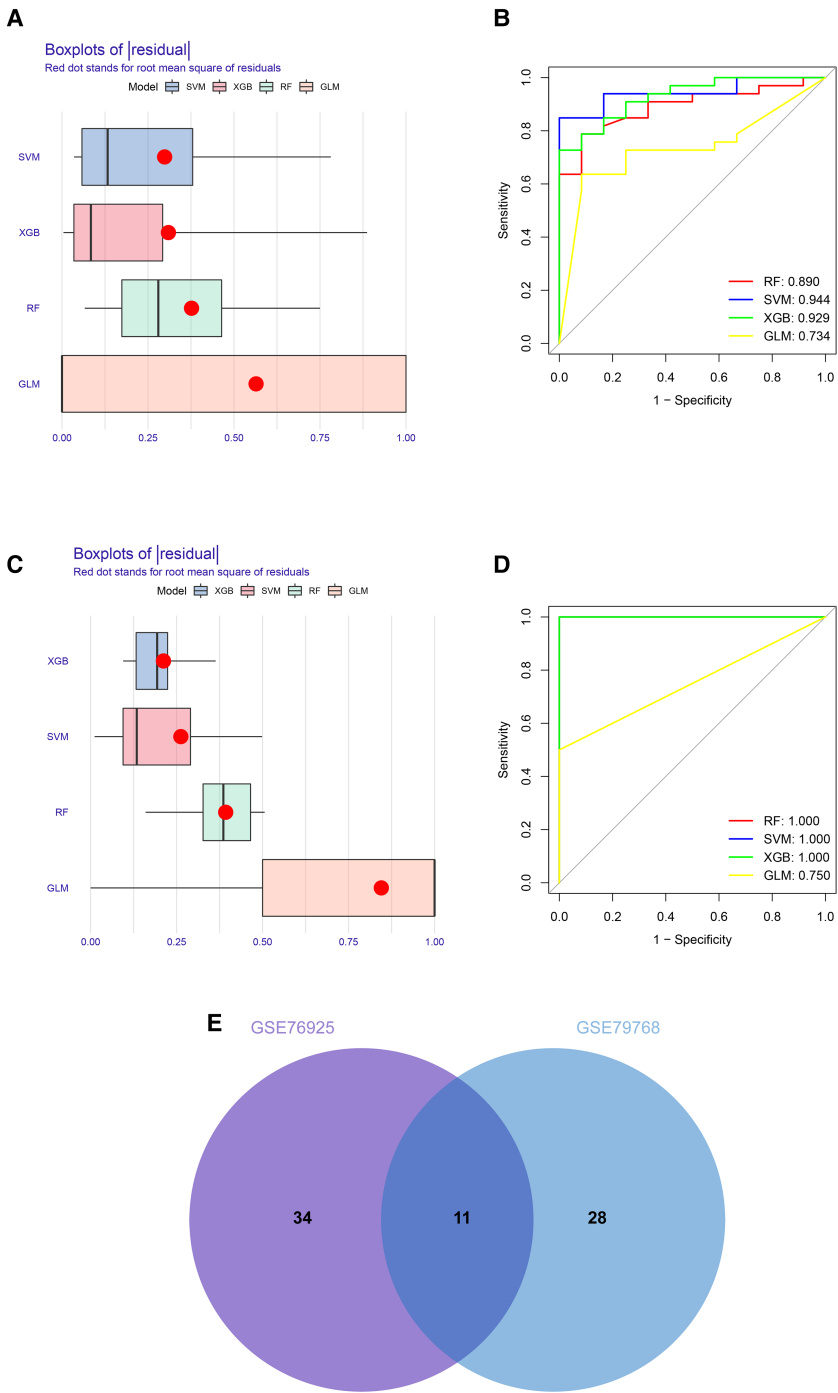
Construction and assessment of machine learning models for COPD and AF. (**A**) Boxplots of the residuals of the COPD. Red dot stands for root mean square of residuals. (**B**) ROC curves for model prediction accuracy in COPD dataset. (**C**) Boxplots of the residuals of the AF. (**D**) ROC curves for model prediction accuracy in AF dataset (**E**) Venn diagram of COPD and AF model prediction genes.

**Figure 5:**
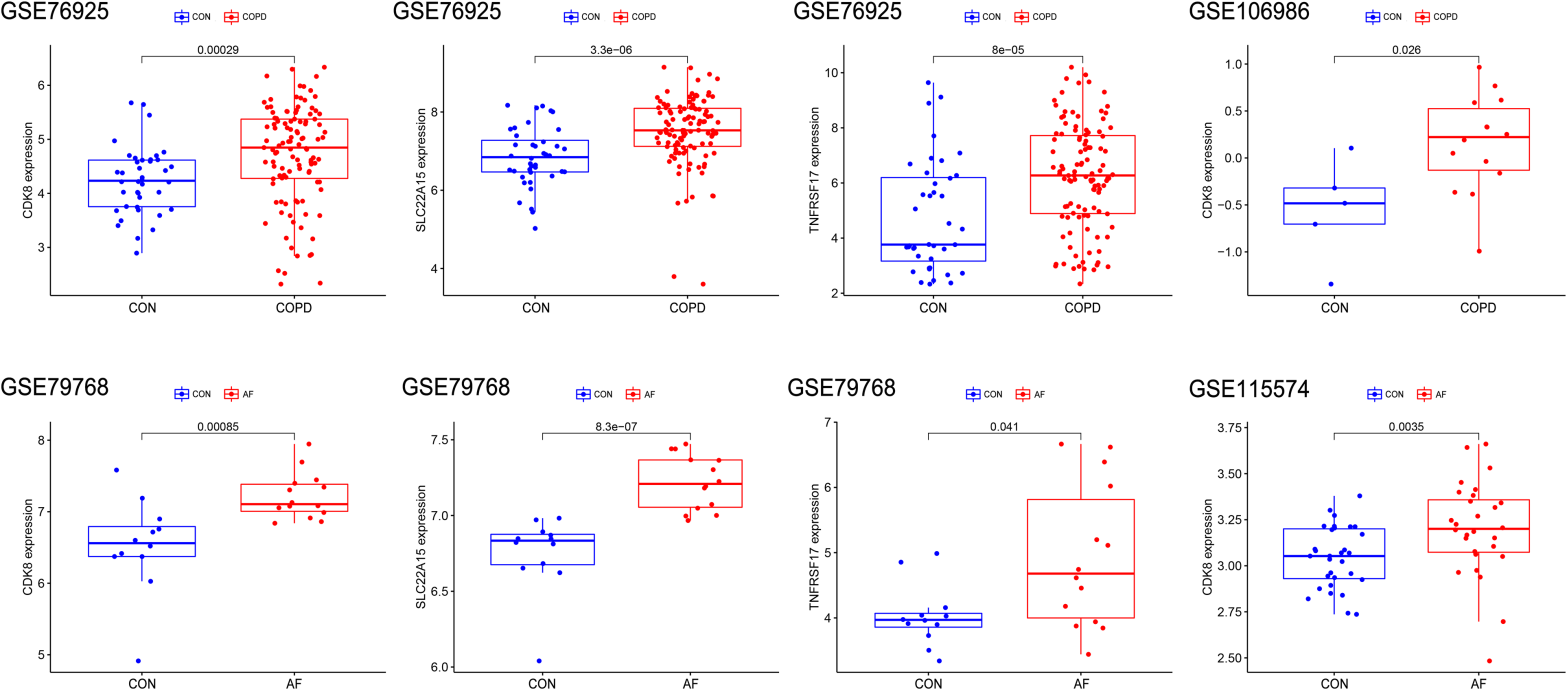
Validation of Hub gene expression levels in training and validation sets. The red box represents the disease group, and the blue represents the control group.

To identify key genes associated with COPD and AF, we constructed models using four machine learning methods and evaluated the models based on residuals and ROC. Box line plots of residuals and ROC are shown in Figures 4A–D. It can be seen that the SVM, XGB and RF models all exhibit similar excellent performance. Therefore, we selected the top 20 genes predicted by RF, SVM, and XGB in each of these two datasets based on the importance scores of the genes assessed by RMSE. Forty-five genes in total in the GSE76925 dataset and 39 in GSE79768, and 11 intersecting genes were obtained after taking the intersection. (Figure 4E, Supplementary Tables S4, S5). After verifying the differential expression and expression trends, three candidate genes, cyclin dependent kinase 8 (*CDK8*), solute carrier family 22 member 15 (*SLC22A15*), and TNF receptor superfamily member 17 (*TNFRSF17*), were finally obtained, and the box plots of differential expression are shown in Figure 5.

### Validation of hub genes and assessment of predictive accuracy

**Figure 6:**
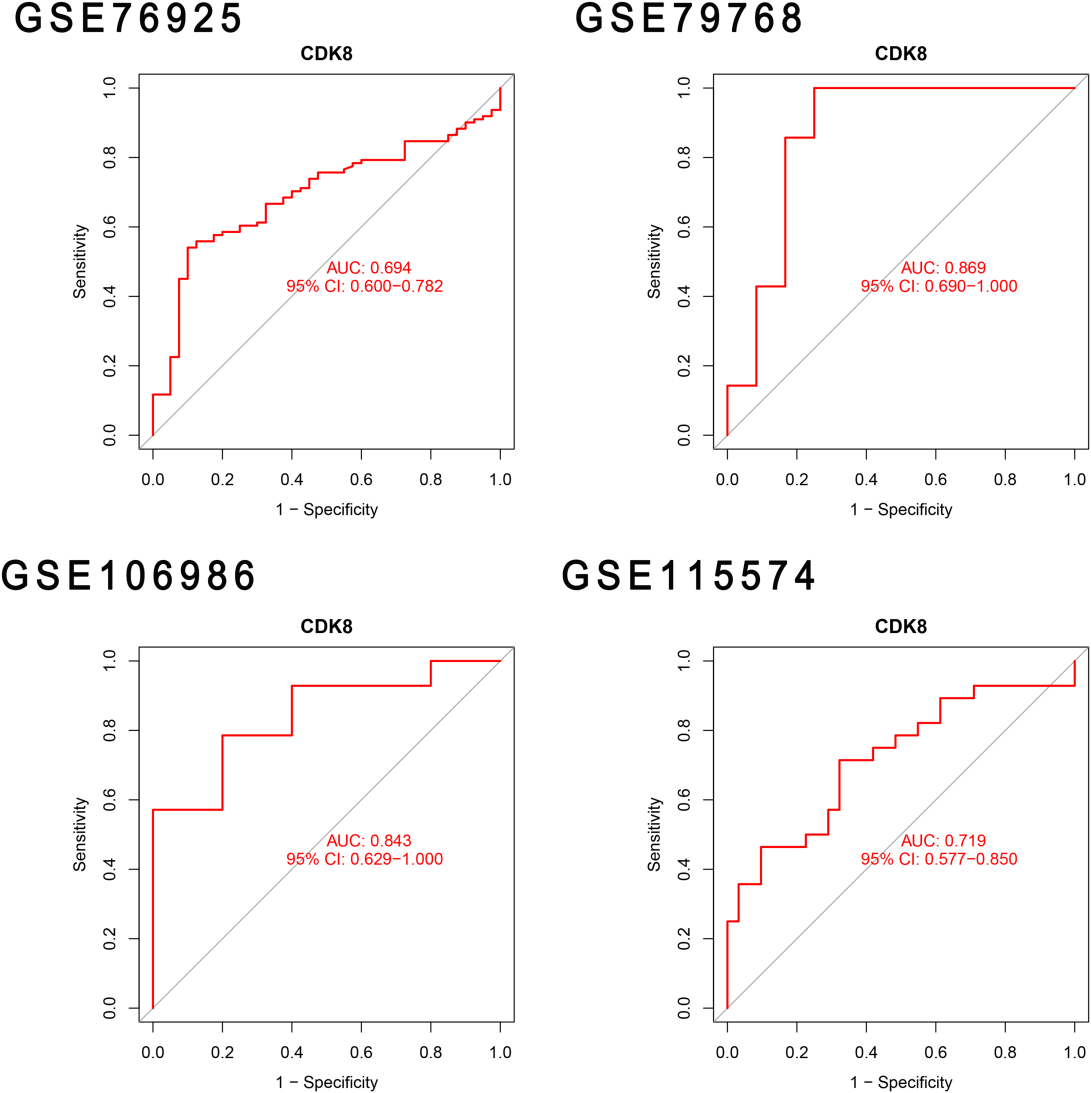
Validation hub genes are used as marker genes.

The differential expression of *CDK8*, *SLC22A15*, and *TNFRSF17* was confirmed in the external validation set. Only *CDK8* showed a significant increase in expression in both the training and validation sets (*P < *0.05) Figure 5. Thus, *CDK8* was finally identified as a hub gene that may be related to AF and COPD. the *CDK8*′*s* ROC curves demonstrated that in all four data sets, the AUC was near to or greater than 0.7 (Figure 6). It is suggested that *CDK8* may be effective for detecting COPD combined with AF.

### Comprehensive analysis of hub genes

**Figure 7:**
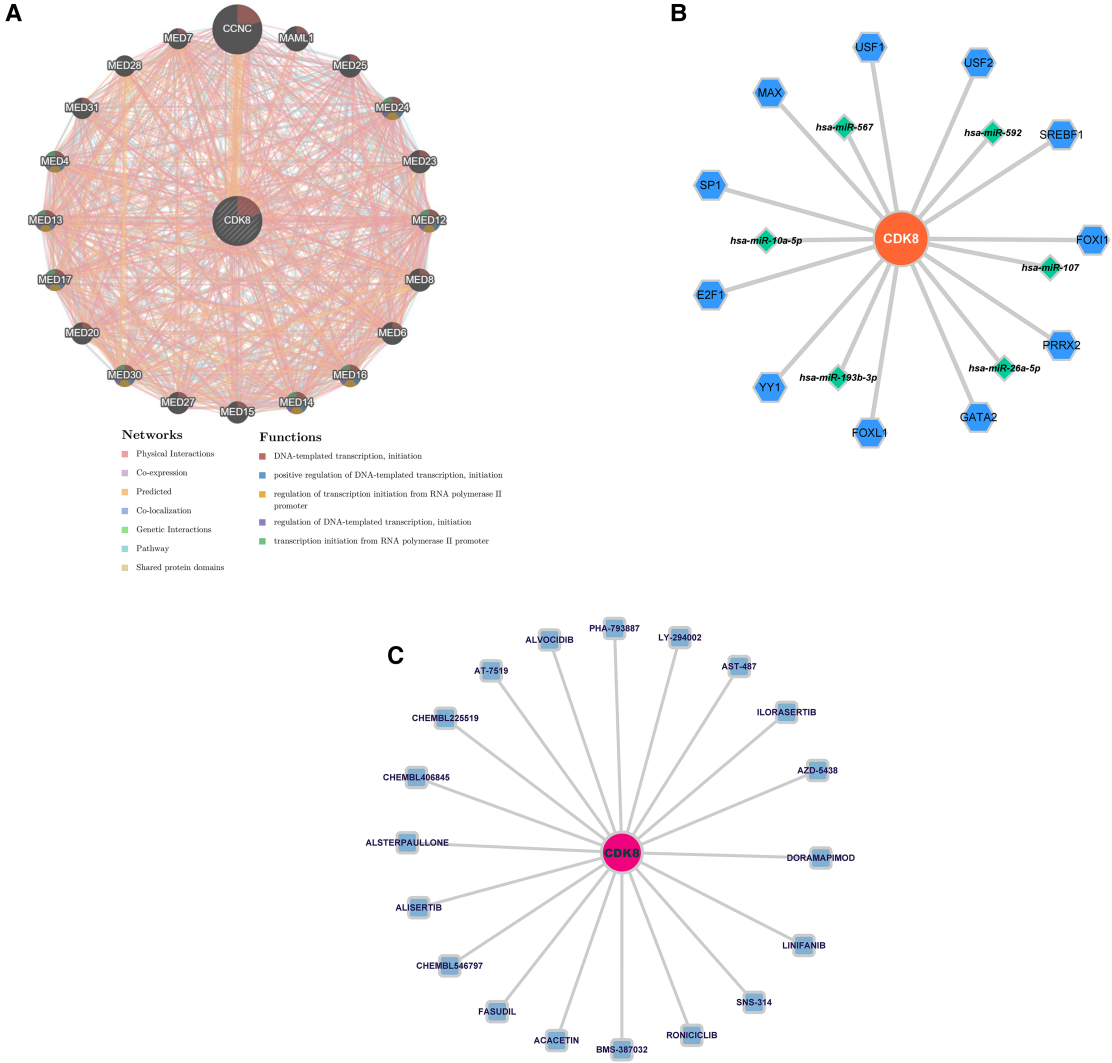
(**A**) Co-expression network of CDK8. (**B**) The “TF-mRNA-miRNA” regulatory network of CDK8. Blue hexagons are TFs; green diamonds are miRNAs. (**C**) Drug prediction for CDK8 based on DIGDB database. Blue squares are potential drugs.

Twenty genes associated with hub genes were predicted by GeneMANIA, along with 1,326 interactions. This suggests a complex interaction between the hub gene and the remaining 20 genes. The functional enrichment results were mainly associated with DNA-templated transcription, positive regulation of DNA-templated transcription, and regulation of transcription initiation from the RNA polymerase II promoter (Figure 7A). The “TF-mRNA-miRNA” regulatory network contains 11 TFs that regulate *CDK8*, and five miRNAs (Figure 7B). DGIDB predicted 20 drugs targeting *CDK8*, including ALVOCIDIB, ACACETIN, and RONICICLIB (Figure 7C).

### Immune cell inﬁltration analysis

**Figure 8:**
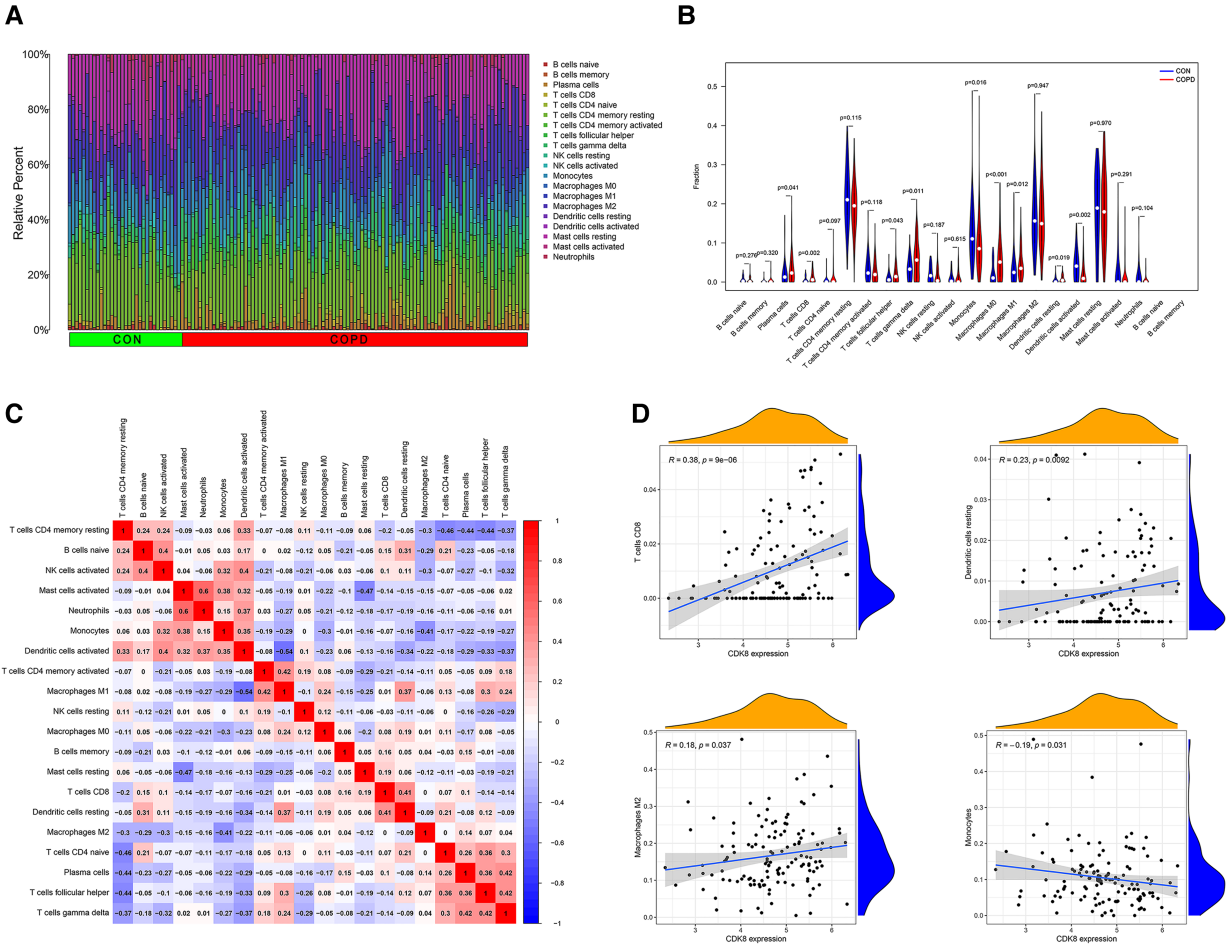
Immune cell infiltration analysis of GSE76925 and immune cell correlation analysis of CDK8. (**A**) The proportion of immune cells in different samples. (**B**) Comparison of immune cell ratios in the COPD and control groups. (**C**) Heat map of correlation analysis between immune cells. (**D**) Analysis of the correlation between CDK8 and immune cells.

By observing the enrichment analysis results, we found that immune-related pathways were significantly enriched, suggesting that immune dysfunction may be involved in the development of AF in COPD patients. Therefore, we performed an immune cell infiltration analysis of gene expression profiles in COPD. Figure 8A shows the ratio of immune cells in the control group to the COPD group. Compared to the control group, the COPD group had higher levels of plasma cells, CD8+ T cells, T follicular helper cells, Gamma-delta (γδ) T cells, macrophage M0, and resting dendritic cells, and lower levels of monocytes, macrophage M1, and activated dendritic cell (Figure 8B). Positive correlations were found between activated mast cells and neutrophils (*r* = 0.60), T cells and plasma cells (*r* = 0.42), and γδ T cells and T follicular helper cells (*r* = 0.42). In contrast, Macrophage M1 and activated dendritic cells were negatively correlated (*r* = −0.54) (Figure 8C). This suggests that patients with COPD have a different immune pattern compared to normal patients and that there are interactions between different types of immune cells. Detailed results of the immune cell infiltration analysis are shown in Supplementary Table S6.

Correlation analysis of *CDK8* and immune cells showed that *CDK8* expression correlated with four immune cell types, positively with CD8+ T cells, resting dendritic cells, and macrophage M2, and negatively with monocytes (Figure 8D).

## Discussion

COPD affects more than 300 million people worldwide and causes approximately 3 million deaths yearly (33). COPD increases the incidence of AF and the risk of subsequent cardiovascular death. Similarly, comorbid AF increases the incidence of ischemic stroke, respiratory failure, and heart failure events in patients with COPD (34–37). The vicious circle relationship between COPD and AF, and the contradiction of pharmacological treatment, makes it urgent to explore the mechanisms of COPD and AF co-morbidity and to find potential therapeutic targets. It is generally believed that COPD-induced structural changes in the heart, increased sympathetic activity, and hypoxia-induced oxidative stress accelerates the development of AF. But recent studies have highlighted the role of immune dysfunction in the pathogenesis of both diseases, finding that immune dysfunction may play a prominent role in the subsequent inflammatory response, cardiac remodeling, structural remodeling, and neural remodeling (11, 38). However, the exact mechanisms have not been fully elucidated. In this context, we performed the first joint analysis of genetic datasets from both diseases to reveal common mechanisms and key biomarkers for the development of both diseases and to reveal changes in immune cells in COPD by immune cell infiltration analysis.

After screening of overlapping genes and the construction of PPI networks, 77 genes with interactions were obtained. They are mainly involved in biological processes such as activation of the immune response, regulation of inflammatory response, leukocyte migration, response to chemokines, immune receptor activity, and signaling pathways such as hematopoietic cell lineage, IgA-producing intestinal immune network, cytokine-cell receptor interactions, and T cell receptors. Combined with the results of GSEA enrichment analysis, we suggest that immune and inflammatory responses are the key mechanisms linking these two diseases, as confirmed by the results of previous studies (39–41). COPD is accompanied by a long-term and persistent chronic inflammatory response in the airways and lung parenchyma, leading to subsequent airway remodeling and destruction of the lung parenchyma (42). After inhalation of smoke or other toxic particles, the immune response is activated, after which macrophages release various cytokines and chemokines, including tumor necrosis factor-alpha (*TNF-α*), interleukin 1 beta (*IL-1β*), interleukin 6 (*IL-6*), C-X-C motif chemokine ligand 1 (*CXCL1*), and C-X-C motif chemokine ligand 8 (*CXCL8*), which attract circulating neutrophils, monocytes, and lymphocytes in the lungs leading to an inflammatory response (39, 43). At the same time, the activation of immune and inflammatory responses is not limited to the lungs, as studies have shown that COPD patients are accompanied by elevated circulating c-reactive protein (*CRP*), *IL-6*, *CXCL8*, and *TNF-α* (44). Changes in immune cells, especially macrophages, and increases in cytokines and chemokines such as *TNF-α, IL-6, IL-1β*, C-X-C motif chemokine ligand 1 (*CXCL1*), and C-X-C motif chemokine ligand 2 (*CXCL2*) have also been observed in AF (45, 46).

In response to these results, we performed an immune cell infiltration analysis in GSE76925, which showed a higher proportion of plasma cells, CD8+ T cells, T follicular helper cells, γδ T cells, macrophage M0, and resting dendritic cells, and a lower proportion of monocytes, macrophage M1, and activated dendritic cells in COPD lung tissue compared with controls. Studies have shown that CD8+ T lymphocytes increase in number and activity in COPD and produce many cytokines such as Interferon gamma (*IFN-γ*) and *TNF-α* (47). γδ T cells are nontraditional T cells, and despite their small number, a study by Murdoch (48) et al. found that γδ T cells increase interleukin 17A (*IL-17*) production during acute allergic airway disease and are involved in disease pathogenesis. Plasma cells are widely present in the connective tissue of the lamina propria of the respiratory tract and participate in the adaptive humoral immune response by synthesizing antibodies in response to the invasion of the respiratory tract (49). Studies have shown that the severity of COPD is positively correlated with the development of tertiary lymphoid organs (TLOs), and IL-21 T-follicular-helper (Tfh)-like cells have been observed in TLOs of COPD patients, suggesting that Tfh may be involved in the formation of TLOs (50, 51). Dendritic cells are critical antigen-presenting cells involved in adaptive immune activation in COPD. However, Givi (52) et al. found that chronic exposure to harmful particles impairs dendritic cells maturation and inhibits antigen-presenting capacity. Macrophages are differentiated from monocytes and play a key role in chronic inflammation in COPD patients. Naive macrophage M0 can be induced to differentiate into M1- and M2-type macrophages under different conditions, with M1-type mainly playing a pro-inflammatory role (53). Interestingly, our study found a lower proportion of M1-type macrophages in the COPD group compared with controls, the reason for which needs to be further investigated. Similarly, immune cells are the primary cell type in the heart, and one study showed that immune cells accounted for 10.4% of all cell types in atrial tissue (54). More dendritic cells were found in the left atrial myocardium of patients with AF compared to those with sinus rhythm. Increased numbers of neutrophils, lymphocytes, and macrophages were also observed in the atrial adipose tissue (55, 56). These findings suggest that immune cell changes in COPD may also be involved in developing AF.

A biomarker, *CDK8*, was identified by a machine learning algorithm and validated in the validation set, and it was significantly upregulated in both COPD and AF groups. *CDK8* is a serine/threonine protein kinase that plays an important role in transcriptional regulation by binding to cell cycle protein C (57). Recent studies have shown that *CDK8* is also involved in the inflammatory response, as Chen et al. (58) found that in response to *TNF-α* stimulation, nuclear factor kappa B (*NF-kB*) and *CDK8* are jointly recruited to the promoters of response genes, driving the expression of *NF-kB* early response genes *CXCL8*, *CXCL2*, and C-X-C motif chemokine ligand 3 (*CXCL3*). In addition, *CDK8* is involved in the activation of hypoxia inducible factor 1 subunit alpha (*HIF1A*) (59). lungs of COPD patients overexpress *HIF1A*, which is associated with hypoxia and inflammatory response (60). Elevated expression of *HIF1A* is also observed during AF, which may be involved in the structural remodeling of the left atrium (61). *CDK8* also highlights potential advantages as a therapeutic target, and *NF-kB* plays an important role in activating immune inflammatory responses in COPD and AF by encoding chemokines and cytokines (62, 63). Transcription of *NF-kB* requires activation of *CDK8*. Studies have shown that reducing *CDK8* activity inhibits *NF-kB*-driven transcription but has no effect on the basal expression of *NF-kB*-regulated genes or promoters (58). This avoids the detrimental effects of *NF-kB* blockers due to reduced *NF-kB* expression levels, making *CDK8* a more promising therapeutic target. In addition, we performed a co-expression analysis of CDK8, “TF-mRNA-miRNA” network construction, and drug prediction. These *CDK8*-associated mRNAs, TFs, and miRNAs also contribute to understanding the *CDK8* association network. At the same time, the results predicted by DGIDB may become new drugs for treating COPD combined with AF. Several studies have shown that inhibitors of *CDK8* have an inhibitory effect on inflammatory immune responses (64, 65). Studies by Schmerwitz et al. (66) have found that ALVOCIDIB (also known as Flavopiridol) is able to against inflammation by effectively blocking the activation of endothelial cells through the inhibition of *NF-kB* consensus promoter activity and thereby disrupting the interaction between inflammatory-induced leukocytes and endothelial cells. This suggests that drugs targeting *CDK8* may be promising for treating COPD combined with AF.

Analysis of the correlation between *CDK8* and immune cell infiltration showed a positive correlation between *CDK8* and CD8+ T cells, resting dendritic cells, and macrophage M2 and a negative correlation with monocytes. Patients with COPD have increased infiltration of CD8+ T cells in lung tissue and produce pro-inflammatory factors such as *TNF-α* (47). The involvement of *TNF-α* in AF involves multiple mechanisms, and *TNF-α* has been shown to disrupt intracellular calcium homeostasis in atrial myocytes by decreasing the expression of T-type calcium channel α1G subunit (TCCA 1G) and sarcoplasmic reticulum Ca-ATPase (SERCA2a) thereby participating in AF (67, 68). In addition, *TNF-α* directly reduces collagen synthesis in cardiomyocytes, enhances matrix metallopeptidase 2 (*MMP-2*) and matrix metallopeptidase 9 (*MMP-9*) activities, promotes collagen breakdown, and exacerbates myocardial fibrosis (69, 70). M2 macrophage infiltration in the lungs of patients with COPD is significantly increased. A study by Kaku (71) et al. showed that M2 macrophages are strongly associated with the severity of COPD and a predicted reduction in expiratory force volume in one second (FEV), suggesting the involvement of M2 macrophages in the development of COPD. Macrophages also secrete elastolytic enzymes, such as *MMP-2* and *MMP-9*, which directly lead to the destruction of lung structures and fibrosis of the atria (72–74). Increased sympathetic nervous system activity triggered by COPD promotes the development of AF (11). Studies have revealed that high catecholamine levels induce sympathetic remodeling by acting on the β1-adrenergic receptors on macrophages to produce inflammatory factors such as *TNF-α*, nerve growth factor (*NGF*), and interleukin 1 alpha (*IL-1*). This process may cause the onset of AF (75). Dendritic cell function in AF and COPD is still being studied. Ravi (76) et al. found that monocyte migration capacity was reduced in COPD patients, which may partially explain the negative correlation between *CDK8* and monocytes. However, the exact mechanism still needs further investigation. In conclusion, our findings provide a new perspective on the pathogenesis of COPD combined with AF from the viewpoint of the inflammatory immune response and suggest a biomarker *CDK8* that could potentially be a therapeutic target.

This study also has several limitations. First, the data in this study were obtained based on the GEO database. Although a dataset containing more samples was selected and validated in an external dataset, the results may be biased due to the different platforms from which they were obtained. Additionally, due to COPD being a heterogeneous disease, there are various phenotypes of COPD, such as small airway-predominant disease, frequent exacerbators, and asthma-COPD overlap, which have different pathophysiological mechanisms that are not completely the same (77). Recent studies have shown that within 90 days of an acute exacerbation of COPD, patients are at a significantly increased risk of an emergency department visit or hospital admission related to AF (78). Another cohort study also found an increased risk of AF in asthmatic patients (79). However, our study was unable to analyze the association between the different phenotypes of COPD and AF separately, so further research is needed to investigate the relationship between these different COPD phenotypes and AF at the genetic level. Secondly, the AUC area of *CDK8* in the COPD training set was less than 0.7, and although the AUC area was improved in the validation set, its value in practical clinical applications still needs further validation. Finally, the specific mechanisms of immune inflammatory response and *CDK8* in COPD with AF and the association between *CDK8* and immune cells need further proof from subsequent *in vivo* and *in vitro* experiments.

## Conclusion

In this study, through bioinformatic analysis, we found that disturbances in immune regulation and subsequent activation of the inflammatory response may have a significant role in COPD combined with AF. Through machine learning algorithms, *CDK8* was finally identified as a key biomarker, and inhibitors targeting *CDK8* may be able to be promising therapeutic agents for COPD combined with AF by inhibiting *NF-kB*-induced immune inflammatory responses.

## Data Availability

The original contributions presented in the study are included in the article/**Supplementary Material**, further inquiries can be directed to the corresponding author/s.'
